# Assessing the significance of cytomegalovirus reactivation in recipients of allogeneic hematopoietic stem cell transplantation: a cohort study

**DOI:** 10.1590/1414-431X2026e15073

**Published:** 2026-04-17

**Authors:** L.P. de Oliveira, E.J.A. Paton, M.F. Giovanardi, A.F. da Silva, L.G. Tavares, C.G. Fernandes, J.O. Dias, R.A. Fabreti-Oliveira

**Affiliations:** 1 Faculdade de Ciências Médicas de Minas Gerais, Belo Horizonte, MG, Brasil; 2 Cancer Center Oncoclínicas, Belo Horizonte, MG, Brasil; 3 Laboratório IMUNOLAB Transplantes, Laboratório de Histocompatibilidade, Belo Horizonte, MG, Brasil

**Keywords:** Cytomegalovirus, Hematopoietic stem cell transplantation, Allogenic transplantation, HLA incompatibilities, Risk factor

## Abstract

Cytomegalovirus (CMV) infection increases morbidity in allogeneic hematopoietic stem cell transplantation (allo-HSCT) recipients. Despite advancements in prevention and treatment, CMV reactivation remains a considerable concern following allo-HSCT. In this study, we aimed to evaluate the impact of CMV reactivation in allo-HSCT recipients on clinical outcomes and patient survival, as well as to identify the risk factors associated with CMV reactivation. This retrospective observational cohort study included data from 67 transplantations performed between 2019 and 2022 at a private hospital in Belo Horizonte, Brazil. Clinical, laboratory, and procedural data were collected. The median age of the patients was 47.0 years, and 52.2% of them were women. The most frequent indication for allo-HSCT was acute myeloid leukemia (28.4%), followed by myelodysplastic syndrome (16.4%). CMV reactivation occurred in 62.7% of the recipients, and the median time to CMV reactivation was 32.5 days. Six patients (9.0%) presented with CMV end-organ disease (83.3% were free of systemic disease); however, when present, gastrointestinal involvement was the most common disease (4.5%). In the multivariate analysis, the risk factors for CMV reactivation included acute lymphoblastic leukemia (P=0.006), mismatched human leukocyte antigen (HLA)-unrelated donors (P=0.002), and graft-*versus*-host disease (P=0.024). One-year overall survival was 57.7%. However, CMV reactivation was not significantly associated with mortality after controlling for confounding factors. CMV reactivation remains a common complication following allo-HSCT, with leukemia type, donor type, and graft-*versus*-host disease affecting the risk of disease recurrence. No direct correlation was observed between CMV recurrence and increased mortality or patient survival.

## Introduction

Cytomegalovirus (CMV) is a *β*-herpesviridae that can establish lifelong latent or persistent infections in susceptible human hosts after primary infection (1,2). CMV infection is prevalent in recipients who underwent allogeneic hematopoietic stem cell transplantation (allo-HSCT), often causing systemic infections that can lead to organ damage and elevate morbidity and non-relapse mortality rates (2,3).

While CMV disease can affect any organ, it predominantly manifests in the gastrointestinal tract and lungs (1,3) and has a greater clinical impact in the first 100 days following allo-HSCT (4,5). The main risk factors for CMV viral reactivation are recipient and donor selection characteristics, transplantation modalities, stem cell source, the presence of graft-*versus*-host disease (GVHD) prophylaxis and treatment, and conditioning regimens (3,6). Thus, regular monitoring of viral load may help identify the optimal period for administering preemptive antiviral therapy (1), thereby reducing the incidence of adverse consequences of CMV reactivation and end-organ disease (1,7).

Without an adequate preventive strategy, approximately 70% of individuals undergoing allo-HSCT experience CMV reactivation (3,8). The detrimental impact of CMV infection on outcomes is evident, with a CMV-positive serostatus before HSCT being considered an independent risk factor for non-relapse mortality (9,10).

In this evolving scenario, conducting a comprehensive assessment of the importance of CMV infection in allo-HSCT recipients and identifying the risk factors for viral reactivation in allo-HSCT are crucial for understanding how specific clinicopathological features affect CMV reactivation and patient outcomes. These insights may be important in guiding clinical decisions to enhance patient care and advance transplant management. This study aimed to evaluate the impact of CMV reactivation in allo-HSCT recipients on clinical outcomes and patient survival, and to identify the risk factors associated with CMV reactivation in this group of patients.

## Material and Methods

### Study design and setting

This retrospective observational cohort study included a convenience sample of 67 recipients who underwent first allo-HSCT between October 2019 and August 2022 at a private hospital in Belo Horizonte, Minas Gerais, Brazil.

The inclusion criteria were individuals of any ethnicity of both sexes and of any age (adults and children) who presented with malignant and non-malignant hematological diseases. The exclusion criterion was the absence of data from medical records or laboratory records; however, no cases were excluded from this study. The study followed patients from the transplant date for at least 100 days, or less for those who died from transplantation-related deaths.

### Patient data

The data were obtained using patient identification codes and then transferred to the Research Electronic Data Capture database, which restricted access to the project's responsible researchers. Data management was performed using electronic data capture tools hosted on virtual servers located at the Ativas^®^ data center, in the city of Belo Horizonte, Minas Gerais, Brazil.

From the patient medical records, the underlying disease and its status, type of allo-HSCT, presence of comorbidities, time between diagnosis and transplantation, conditioning regimen, source of hematopoietic stem cells, immunosuppressors used after HSCT, prophylactic treatment received, date of CMV reactivation diagnosis, treatment received, and the presence or absence of GVHD were identified. The exposure was CMV reactivation, and the outcomes included base disease recurrence, overall survival, and mortality.

### Detection of CMV reactivation

Detection of CMV reactivation was based on the quantification of viral load in plasma using the Xgen Master CMV assay (Mobius Life Science, Brazil). According to our institutional protocol, a viral load exceeding 500 IU/mL by PCR was used as the threshold to define CMV reactivation and initiate preemptive antiviral therapy.

For the purposes of this study, we distinguished between CMV reactivation and clinical CMV infection. CMV reactivation was defined solely as the detection of CMV DNA in plasma by PCR at levels exceeding 500 IU/mL, regardless of clinical symptoms. Clinical CMV infection was defined as the presence of CMV-associated end-organ disease, identified through clinical evaluation and confirmed by supporting laboratory or imaging findings. This included, but was not limited to, manifestations such as gastrointestinal disease, pneumonitis, or other organ-specific involvement attributable to CMV.

### GVHD severity classification

GVHD severity was classified into two main categories: mild (Grades I and II) and severe (Grades III and IV). Mild GVHD included Grade I, characterized by a rash involving less than 25% of the body surface, serum bilirubin levels between 2-3 mg/dL, and mild diarrhea (less than 500 mL/day), and Grade II, with a rash involving 25-50% of the body surface, serum bilirubin levels between 3-6 mg/dL, and moderate diarrhea (500-1000 mL/day). Severe GVHD included Grade III, characterized by a rash involving more than 50% of the body surface, serum bilirubin levels between 6-15 mg/dL, and severe diarrhea (>1000 mL/day) and Grade IV, with a bullous or exfoliative rash resembling severe burns, serum bilirubin levels above 15 mg/dL, and intense abdominal pain, ileus, or massive diarrhea (>1500 mL/day).

### Prophylactic and therapeutic strategies

None of the patients in the study received prophylaxis with letermovir owing to the unavailability of authorization by healthcare providers. Treatment for CMV reactivation or infection involved the use of ganciclovir, valganciclovir, or both at different times in the same patient.

Intravenous ganciclovir was uniformly adopted as the first-line induction therapy for CMV reactivation, given its established role and accessibility within our clinical practice. Valganciclovir was primarily reserved for maintenance therapy following initial viral load reduction achieved with ganciclovir.

Treatment with ganciclovir was carried out at a dose of 5 mg/kg every 12 h for 14 days or until the PCR result was negative, whichever occurred later. Maintenance therapy was given for 1 week at a dose of 6 mg/kg per day. Valganciclovir was administered at a dose of 900 mg every 12 h for 14 days or until the PCR result was negative, whichever occurred later. Maintenance therapy was administered for one week at 900 mg once a day.

Patients who received ganciclovir and valganciclovir were administered ganciclovir for the first 14 days, followed by maintenance therapy with valganciclovir. Therapeutic response failure to CMV treatment was defined as a reduction of less than 1 log_10_ in CMV PCR viral load after two weeks of appropriate antiviral therapy, or as stabilization or any increase in viral load during continuous antiviral treatment, even after an initial decline. Conversely, treatment response was considered when the CMV viral load decreased by more than 1 log_10_ after more than two weeks of antiviral therapy. Additionally, progressive end-organ disease despite adequate treatment was also considered indicative of treatment failure.

### Transplant-related mortality

Transplant-related mortality was defined as death from complications associated with HSCT, including infections, GVHD, medication toxicity, bleeding, or graft failure. These events primarily occurred within the first 100 days following allo-HSCT. Deaths attributed to disease relapse or progression were not included in this definition.

### Ethical considerations

This study was performed in compliance with the Declaration of Helsinki and was approved by the Research Ethics Committee of the Faculty of Medical Sciences of Minas Gerais, Belo Horizonte, Minas Gerais, Brazil (number 5.894.381/2023).

### Statistical analysis

Qualitative variables are reported as frequencies and percentages. Numerical variables are reported as minimum, maximum, mean, standard deviation, median, first (Q1), and third (Q3) quartiles. For all variables, a valid number of non-missing data (n) is presented. No imputation of missing information was used. The association between qualitative variables was assessed using Fisher's exact test. Overall survival and time to CMV reactivation were assessed using the Kaplan-Meier (KM) method, and survival curves were compared using the log-rank test. The risk factors for CMV reactivation were assessed using the Cox model. All variables were included in a full model, and using the backward strategy, the final model was obtained, in which significant variables were maintained. To evaluate the effect of CMV reactivation as a risk factor for death, the Cox model was also used. The results of the Cox models are presented as hazard ratios (HR) and respective 95% confidence intervals (CI), and the proportionality of risks was assessed using Schoenfeld residuals. Statistical analyses were performed in the RStudio program version 2023.12.0, using R version 4.3.2, and P<0.05 was considered significant. For statistical purposes, donor type was categorized as matched related donor (MRD), matched unrelated donor (MUD), mismatched unrelated donor (MMUD), and mismatched related donor (MMR), with the latter including all haploidentical transplants.

## Results

### Patient demographics and clinical characteristics

The sample consisted of 67 patients (52.2% women) with a median age of 47.0 years (Q1: 27.5; Q3: 60.5). Regarding educational level, 31.3% had complete higher education, 31.3% had complete secondary education, and 10.4% had incomplete primary education. The percentage of patients from Belo Horizonte was 31.3%, whereas 65.7% were from other cities in Minas Gerais. Among the donors, 61.2% were men, with a median age of 36.0 years (Q1: 29.5; Q3: 45.2). Table 1 shows the clinical characteristics of the transplants, which had a myeloablative conditioning regimen in 62.7% of the cases. The main source of stem cells was peripheral blood in 80.6% of the cases.

**Table 1:** Characterization of patients undergoing allogeneic hematopoietic stem cell transplantation at a private hospital in Brazil between August 2019 and August 2022.

Variables	N	Statistics
Time between diagnosis and transplant (months)	67	
Min/max		1.2/385.9
Median [Q1; Q3]		10.0 [6.3; 18.5]
Mean (SD)		24.6 (51.3)
Underlying disease	67	
AML		19 (28.4%)
Stage	19	
Total remission		16 (84.2%)
Partial remission		2 (10.5%)
No remission - active disease		1 (5.3%)
Myelodysplastic syndrome		11 (16.4%)
Stage	11	
Low risk		1 (9.1%)
Intermediate risk		2 (18.2%)
High risk		3 (27.3%)
Unclassified		5 (45.5%)
ALL		12 (17.9%)
Stage	12	
Total remission		11 (91.7%)
Partial remission		1 (8.3%)
Non-neoplastic diseases^†^		7 (10.4%)
Stage	7	
Unclassified stage		7 (100.0%)
Myelofibrosis		5 (7.5%)
Stage	5	
Progressive disease		5 (100.0%)
Hodgkin's lymphoma		4 (4.8%)
Stage	4	
Complete response		1 (25.0%)
Partial response		3 (75.0%)
Others		9 (10.8%)
Unclassified stage	9	9 (100.0)
Comorbidities^‡^	67	33 (50.7%)
Hypothyroidism		12 (17.9%)
Hypertension		10 (14.9%)
Others^§^		24 (35.8%)
Type of donor	67	
MMR		25 (37.3%)
MRD		29 (43.3%)
MMUD		1 (1.5%)
MUD		12 (17.9%)
Transplant conditioning regimen	67	
Myeloablative		42 (62.7%)
Non-myeloablative		25 (37.3%)
Stem cell source	67	
Bone marrow		13 (19.4%)
Peripheral blood		54 (80.6%)
Presence of GVHD^‡^	67	42 (62.7%)
Acute disease	42	36 (85.7%)
Mild		15 (41.7%)
Severe		10 (27.8%)
Unclassified		11 (30.6%)
Chronic disease	42	13 (31.0%)
Mild		7 (53.8%)
Severe		2 (15.4%)
Unclassified		4 (30.8%)
Use of corticosteroids for GVHD treatment	67	31 (46.3%)
GVHD prophylaxis regimen^†^		
Calcineurin inhibitors + Methotrexate	67	4 (6.0%)
ATG + calcineurin inhibitors + methotrexate	67	37 (55.2%)
Post-cyclophosphamide + calcineurin inhibitors + mycophenolate	67	23 (34.3%)
ATG + post-cyclophosphamide + calcineurin inhibitors + mycophenolate	67	2 (3.0%)
Post-cyclophosphamide + calcineurin inhibitors	67	1 (1.5%)
Relapse	67	17 (25.4%)
Time between transplantation and relapse (months)	17	
Min/max		1/33
Median [Q1; Q3]		6.0 [2.0; 9.0]
Mean (SD)		7.6 (8.2)
Bone marrow engraftment	67	62 (92.5%)
Time to bone marrow engraftment (days)	62	
Min/max		10.0/46.0
Median [Q1; Q3]		14.0 [13.0; 17.8]
Mean (SD)		15.9 (5.7)
Follow-up time (months)	67	
Min/max		0/42.0
Median [Q1; Q3]		13.0 [3.5; 22.5]
Mean (SD)		14.8 (12.2)

^†^Non-neoplastic diseases are sickle cell disease (n=1) and marrow aplasia (n=7). ^‡^Multiple responses are possible. ^§^Other comorbidities with more than one citation: depression (n=4), obesity (n=4), splenectomized (n=3), deep vein thrombosis (n=3), breast cancer (n=3), smoking (n=3), alcoholism (n=2), chronic obstructive pulmonary disease (n=2), previous pulmonary aspergillosis (n=2), hypercholesterolemia (=2). Q1: first quartile; Q3: third quartile; SD: standard deviation; AML: acute myeloid leukemia; ALL: acute lymphoblastic leukemia; ATG: anti-thymocyte globulin; GVHD: graft-*versus*-host disease; MMR: mismatched related donor; MRD: matched related donor; MMUD: mismatched unrelated donor; MUD: matched unrelated donor. Time to bone marrow engraftment: neutrophil engraftment.

### Clinical course and outcomes

Among the 67 patients who underwent allo-HSCT, 50.7% died, and 55.9% of these deaths were transplant-related. The median overall survival time for patients was 20.2 months, and the 12-month survival rate was 57.7% (95%CI: 46.9-71.0%). The median follow-up time was 13.0 months (Q1: 3.5; Q3: 22.5). Disease relapse was observed in 25.4% of the patients, and the median time between transplantation and relapse was 6.0 months (Q1: 2.0; Q3: 9.0) (Table 1).

**Figure 1:**
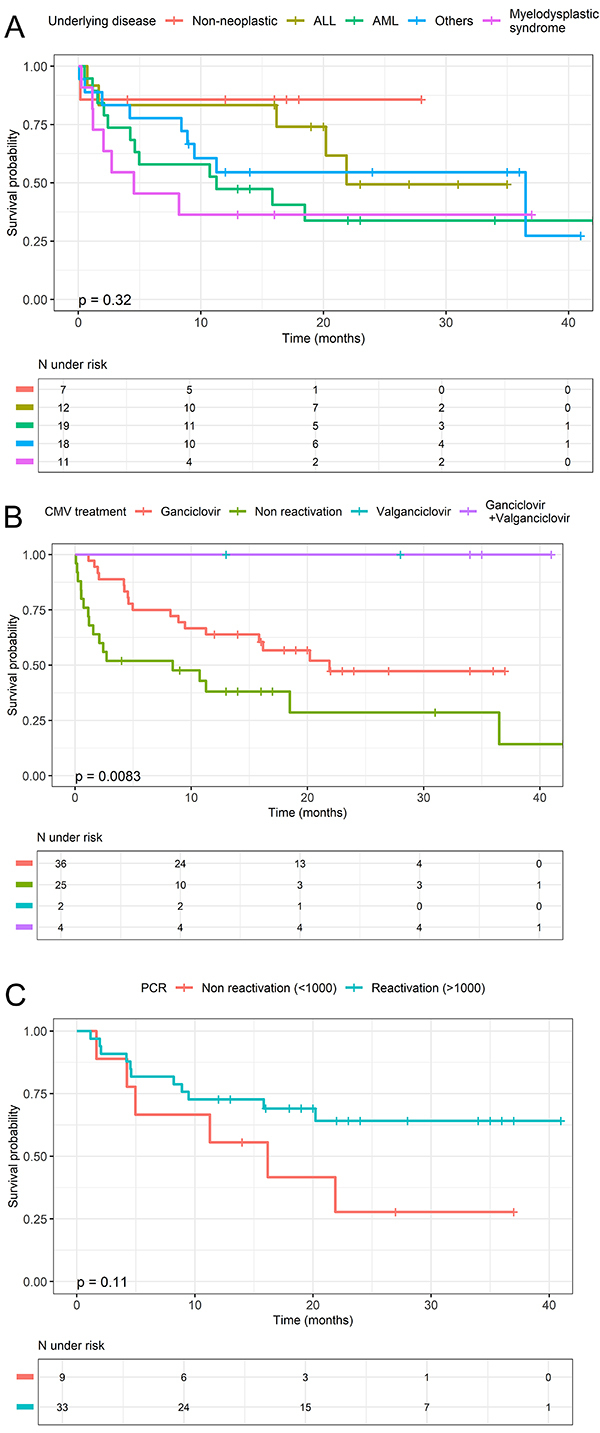
Kaplan-Meier estimates of overall survival in patients post allo-HSCT. **A**, Survival curves categorized by underlying disease (P=0.32). **B**, Survival curves based on CMV treatment regimen (P=0.0083). **C**, Survival curves based on CMV reactivation status (P=0.11). The number of patients at risk over time is indicated in the tables below each graph. CMV: cytomegalovirus; PCR: polymerase chain reaction; ALL: acute lymphoblastic leukemia; AML: acute myeloid leukemia.

The mortality rate among patients with a clinical CMV infection was 50.0%, whereas among those who had reactivated CMV but did not have a clinical infection, the mortality rate was 38.9% (P=0.949). Based on the underlying disease, the mortality rates were as follows: 63.2% for patients with acute myeloid leukemia (AML), 41.7% for patients with acute lymphoblastic leukemia (ALL), 63.6% for patients with myelodysplastic syndrome, 50.0% for patients with other malignancies, and 14.3% for patients with non-neoplastic diseases (P=0.210) (Figure 1A).

Among patients treated for GVHD using ganciclovir alone, the mortality rate was 47.2%. There were no deaths among patients treated with ganciclovir plus valganciclovir or valganciclovir alone (P=0.005) (Figure 1B). In the group of patients with a viral load of >1000 IU/mL (determined using PCR), the mortality rate was 33.3%, whereas in the group of patients with a viral load of ≤1000 IU/mL (determined using PCR), the mortality rate was 66.7% (P=0.124) (Figure 1C).

Among the cases in which bone marrow engraftment occurred, all deaths occurred after engraftment, with 48.3% of deaths related to transplantation. All five patients who experienced graft failure died, of which three (60.0%) had graft failure as the cause of death.

There were no significant differences in survival between the PCR≤1000 and >1000 strata (P=0.100), between adult and pediatric populations (P=1.000), between patients with malignant and non-malignant underlying diseases (P=0.200), or between various underlying diseases (P=0.300).

### Characterization of CMV reactivation and infection

All the 42 cases of CMV reactivation had PCR-confirmed viral loads exceeding 500 IU/mL. The overall incidence of CMV reactivation was 62.7%, with a median onset at 32.5 days post-allo-HSCT (95%CI: 26.2-40.8). Treatment predominantly involved ganciclovir, used in 95.2% of cases, while valganciclovir was administered in 14.3% of cases. Additionally, 6% of patients received both drugs sequentially at different stages of their treatment (Table 2).

**Table 2:** Characterization of cytomegalovirus (CMV) samples from patients undergoing allogeneic hematopoietic stem cell transplantation at a private hospital in Brazil between August 2019 and August 2022.

Variables	N	Statistics
CMV serology	67	
D-/R+		10 (14.9%)
D+/R-		2 (3.0%)
D+/R+		55 (82.1%)
CMV Reactivation	67	42 (62.7%)
Time of CMV reactivation post-transplant (days)	42	
Min/max		11.0/261.0
Median [Q1; Q3]		32.5 [26.2; 40.8]
Mean (SD)		41.1 (38.9)
Treatment	67	
Ganciclovir		40 (59.7%)
Valganciclovir		6 (9.0%)
Clinical CMV disease	67	
Absent		61 (91.0%)
GI tract involvement		3 (4.5%)
Respiratory tract involvement		2 (3.0%)
Other		1 (1.5%)
CMV pneumonitis and hepatitis		1 (100.0%)
Preemptive CMV treatment response	42	37 (88.1%)

Q1: first quartile; Q3: third quartile; SD: standard deviation; GI: gastrointestinal.

A response to preemptive treatment for CMV was observed in 88.1% of cases (Table 2). Transplant conditioning regimen, type of allo-HSCT, and GVHD prophylaxis were not associated with CMV reactivation (Table 3).

**Table 3:** Transplant conditioning regimen, type of allogeneic transplantation, and graft-*versus*-host disease prophylaxis regimen according to cytomegalovirus (CMV) reactivation in patients undergoing allogeneic hematopoietic stem cell transplantation at a private hospital in Brazil between August 2019 and August 2022.

Variable	CMV reactivation		P
	No (n=25)	Yes (n=42)	
Transplant conditioning regimen			0.383
Myeloablative	14 (33.3%)	28 (66.7%)	
Non-myeloablative	11 (44.0%)	14 (56.0%)	
Type of allogeneic transplantation			0.709
MMR	8 (32.0%)	17 (68.0%)	
MRD	11 (37.9%)	18 (62.1%)	
MMUD	0 (-)	1 (100.0%)	
MUD	6 (50.0%)	6 (50.0%)	
GVHD prophylaxis regimen^†^			
Calcineurin inhibitors + methotrexate	2 (50.0%)	2 (50.0%)	0.626
ATG + calcineurin inhibitors + methotrexate	15 (40.5%)	22 (59.5%)	0.544
Post-cyclophosphamide + calcineurin inhibitors + mycophenolate	7 (30.4%)	16 (69.6%)	0.400
ATG + post-cyclophosphamide + calcineurin inhibitors + mycophenolate	1 (50.0%)	1 (50.0%)	1.000
Post-cyclophosphamide + calcineurin inhibitors	0 (-)	1 (100.0%)	1.000

^†^Multiple responses are possible. P-values were calculated using Fisher's exact test. Percentages calculated per row. GVHD: graft-*versus-*host disease; ATG: anti-thymocyte globulin; MMR: mismatched related donor; MRD: matched related donor; MMUD: mismatched unrelated donor; MUD: matched unrelated donor.

A higher risk for CMV reactivation was observed in patients with acute lymphoblastic leukemia (ALL) compared with patients with acute myeloid leukemia (AML) (HR 3.49 [1.43; 8.54], P=0.006), in patients who underwent transplantation from MMUD compared with MRD (HR 82.14 [4.99; 1,352.89], P=0.002), and in patients with acute or chronic GVHD (HR 2.44 [1.13; 5.27], P=0.024). CMV reactivation status and CMV serology of the transplant donors and recipients were not associated (P=0.469).

### Evaluation of CMV reactivation as a risk factor for death

CMV reactivation controlled for underlying disease, donor type, and presence of GVHD was not significantly associated with death (HR 0.55 [0.24; 1.25], P=0.140). The model did not exhibit multicollinearity.

### Distribution of GVHD prophylaxis regimens according to the type of allo-HSCT

Regarding the distribution of prophylaxis regimens for GVHD based on the type of allo-HSCT, 92.0% of mismatched related transplants utilized the regimen comprising post-treatment cyclophosphamide with calcineurin inhibitors and mycophenolate. For MRD, 82.8% received a regimen consisting of anti-thymocyte immunoglobulin (ATG), calcineurin inhibitors, and methotrexate, which was also used for one case of MMUD and all of MUD transplants.

## Discussion

The reactivation of CMV is a frequent concern following allo-HSCT (6,11). In the absence of CMV prophylaxis and depending on the transplantation settings, the incidence of CMV reactivation after allo-HSCT, especially among CMV-seropositive transplant recipients, can be as high as 80% (9,12,13). Importantly, CMV infection has been shown to decrease survival rates following HSCT (4,14). Moreover, myeloablative regimens containing T-cell-depleting agents, such as alemtuzumab and ATG, raise the risk of CMV reactivation and end-organ disease (15-17). In this study, we addressed several important insights regarding CMV reactivation in allo-HSCT recipients. For a comprehensive examination, we included 67 patients who underwent allogeneic HSCT for various hematological disorders, mainly malignancies.

We found that CMV reactivation rates were high, occurring in 62.7% of the cases during the post-transplant follow-up period. Although Styczynski (14) documented a lower recurrence rate of 37%, research conducted in other developing countries has reported CMV reactivation frequencies similar to our observations. For instance, studies in South Asia showed CMV reactivation frequencies of 66.1% (18), consistent with the tendency for CMV seroprevalence to be highest in South America, Africa, and Asia, and lowest in Western Europe and the United States (19). In a recent systematic review from 2023, Cho et al. (20) reported that in the Asia-Pacific, Latin America, and Middle East regions, the CMV recurrence rate ranged from 19.8 to 37.9% in allo-HSCT recipients, indicating a wide variation of CMV reactivation in particular populations and time points, stressing the importance of effective comprehension, prevention, and treatment measures. Variability in reactivation rates is due to population differences in heterogeneous CMV serostatuses before transplantation, conditioning protocols, and diagnostic laboratory techniques (21,22).

Despite the elevated reactivation rates observed, only 9.0% of allo-HSCT recipients demonstrated clinical CMV disease, and a favorable response to ganciclovir or valganciclovir as preemptive treatment was noted in 88.1% of cases. When clinical disease occurs, the majority present with gastrointestinal or respiratory tract involvement. Gastrointestinal disease is the most prevalent form of CMV-related illness in allo-HSCT recipients. Following the introduction of preemptive or prophylactic therapies, CMV pneumonia has become less common. In rare cases, CMV can impact other organs, resulting in conditions such as hepatitis and retinitis, often manifesting as a late-stage complication of the disease, as observed in only one of our patients. Furthermore, our findings are consistent with those across Latin America, where reactivation and disease incidences were 69 and 11%, respectively (23). Specifically, in the Colombian population, even higher CMV disease rates were reported, reaching 16% post-allo-HSCT (21).

Numerous studies have identified risk factors for CMV reactivation (14,24,25). Among these, the primary risk factors consistently reported in the literature include transplant recipient CMV-positive serostatus (R+), acute or chronic GVHD, and unrelated donor or mismatched donor transplant (9,14,26). In our analysis, transplant recipients who exhibited a positive CMV serostatus (both R+/D+ and R+/D-) before allo-HSCT demonstrated a 62.7% CMV reactivation rate. Seropositive recipients who received grafts from seronegative donors are at the highest risk (5,15), likely due to delayed reconstitution of functional CMV-specific T-cell responses following ablation (15,27). However, our data regarding CMV reactivation and serostatus were not associated (P=0.469), which could be attributed to the relatively small sample size, encompassing certain categories of donors and transplant recipients (especially a few R-cases).

The pathogenesis of CMV infection and disease is multifaceted, involving intricate interactions between CMV and the immune system (28). Our study delved into the impact of transplant compatibility on CMV recurrence risk within the studied population. We found that the risk of CMV recurrence was six times higher in partially HLA-matched unrelated transplants than in HLA-MRD (HR 6.07 [1.20; 30.54], P=0.029), a trend consistent with previous investigations (14). Indeed, it has been noted that CMV reactivation tends to be more prevalent in instances of HLA mismatching, with HLA mismatching also being linked to a notable increase in GVHD and graft failure (29).

In this scenario, CMV infection and GVHD are significant complications following allo-HSCT (18,22,24,30). In this study, acute or chronic GVHD was observed in 62.7% of transplants. Patients diagnosed with GVHD showed a nearly three-fold higher risk of CMV reactivation (HR 2.44 [1.13; 5.27], P=0.024), consistent with previous findings (14). The association between CMV and GVHD is believed to be reciprocal, and multiple studies have demonstrated that GVHD and its treatment increase the susceptibility to CMV replication (18,30-32). Moreover, the delay in immune reconstitution due to GVHD and CMV-encoded proteins with immunosuppressive and proinflammatory properties, such as IL-6, may contribute to the initiation of GVHD (11,15,30).

Furthermore, while various minor risk factors have been documented in the literature, including advanced age, stem cell source, intensity and type of conditioning regimen, T-cell depletion, immunosuppressive treatment, immune recovery after HSCT, and use of steroids (3,14), our study did not clearly identify these additional features as significant risk factors for CMV reactivation. In our study, a higher risk of CMV reactivation was observed in patients with ALL than in those with AML (HR 3.49 [1.43; 8.54], P=0.006). We speculate that the use of ATG might offset this benefit (33). However, additional studies investigating this mechanism are necessary for confirmation.

Our results indicated a 12-month overall survival rate of 57.7% after transplantation. Interestingly, our analysis revealed that CMV reactivation was not significantly associated with mortality when factors, such as underlying disease, transplant type, and the presence of GVHD were controlled (HR 0.55 [0.24; 1.25], P=0.140). These findings challenge previous assumptions regarding a direct association between CMV reactivation and mortality in allo-HSCT recipients. CMV reactivation is widely recognized as one of the leading causes of morbidity and mortality following allo-HSCT (25). Previous cohorts have demonstrated that CMV reactivation correlates with a reduction in OS of approximately 17.1% (18). Conversely, other investigations have suggested that CMV infection does not have a significant effect on one-year overall mortality (18).

Although CMV reactivation has traditionally been viewed as a significant contributor to adverse post-transplantation outcomes, our study suggests a more nuanced relationship that may be influenced by various confounding factors, such as the high frequency of CMV reactivation observed in our samples. The viral load considered positive for CMV reactivation varies across large centers due to organizational, financial, and technical differences, with most centers considering a value above 1000 IU/mL to be a CMV reactivation (34). To investigate the impact of different diagnostic thresholds, we re-analyzed mortality using a PCR cutoff value above 1000 IU/mL instead of 500 IU/mL. In this scenario, the mortality rate for patients above the threshold (PCR >1000 IU/mL) was 35.3%, while for those below it (PCR ≤1000 IU/mL) it was 58.3% (P=0.190), showing that the difference in the value determined for reactivation had no impact on the patients' overall survival. The lack of scientific evidence demonstrating the superiority of specific cutoff points is reflected in the variation in reactivation diagnosis among different centers.

Moreover, we must recognize that mortality in patients with HSCT is not solely due to viral infection, but also includes GVHD, immunosuppression, and often the development of severe bacterial infection (21). Regardless of mortality considerations, the morbidity associated with CMV is significant, as CMV disease affects a notable proportion of patients with HSCT, consistent with our findings (5,14,31).

Preemptive treatment is the main strategy for preventing CMV infection post-HSCT, beginning promptly upon early CMV detection (31). The primary anti-CMV medications are intravenous ganciclovir or oral valganciclovir, whereas foscarnet and cidofovir are secondary options for resistant or refractory CMV cases (31). Antiviral agents have achieved CMV elimination in up to 70% of cases, significantly reducing CMV disease incidence. However, intravenous ganciclovir may require hospitalization, posing logistical challenges and escalating treatment costs. Moreover, routine preemptive therapy can cause significant side effects, such as granulopenia/agranulocytosis and renal dysfunction (31,35).

Especially in high-risk patients, such as recipients of HLA-MMR and HLA-MMUD transplants, CMV-seropositive recipients, patients receiving myeloablative regimens, and those with GVHD (15), newer prophylactic agents, including letermovir, which have favorable safety profiles, have the potential to redefine chemoprophylaxis as the standard of care for preventing CMV reactivation. Previous treatments were limited by significant toxicity (31). As a result of therapeutic advances against CMV infection, the incidence of CMV disease in the early stages of HSCT has declined, with the current morbidity rate around 3% (31,36). Letermovir is a notable breakthrough because of its favorable safety profile and effectiveness in reducing the incidence of resistant or refractory CMV disease without causing myelotoxicity or nephrotoxicity (15,37).

Healthcare providers who treat allo-HSCT recipients must use evidence-based strategies to manage potential CMV infections in high-risk populations. Understanding CMV recurrence risk factors can enhance the customization of treatments for individual patients, improving their chances of avoiding end-organ disease and helping to develop strategies to minimize CMV-related complications post-transplantation. While numerous risk factors linked to CMV reactivation are noteworthy (9,12,13,15,17), our findings particularly highlight the subgroup of patients with ALL, as well as those with HLA mismatch and GVHD.

The relationship between CMV serostatus and mortality is more complex than previously thought, and these inconsistencies have been previously documented (38). There is an ongoing debate regarding the influence of CMV replication on patient survival post-allo‐HSCT due to high heterogeneity across studies. Several challenges contribute to this controversy, such as the detection method and duration of CMV monitoring, adjustment for confounding factors, such as HLA matching, donor type and stem cell source, and the potential impact of preemptive therapy on mortality (38).

Our study had some limitations. First, the retrospective nature of the study restricted its ability to establish direct causal relations. Furthermore, the small sample size from a single center may not reflect the diversity of patients undergoing HSCT, which may influence the generalizability of the results. In addition, although subgroup analyses were performed, the study was not originally designed or powered to detect differences within these subgroups, and the results should therefore be interpreted with caution. Moreover, CMV- and host-specific genetic factors that may influence reactivation and survival were not explored in this study. Investigations with larger cohorts and longitudinal follow-ups are needed to explore this matter, along with examining other minor risk factors. Furthermore, none of the patients received letermovir prophylaxis, a medication shown to reduce CMV reactivation. Investigating novel preventive and therapeutic interventions for CMV reactivation is crucial for improving patient care and transplant outcomes and underscores the importance of integrating donor characteristics and risks into a comprehensive evaluation tailored to individual recipient profiles.

Future research should focus on larger multicenter cohorts to enhance statistical power and generalizability, particularly in Brazilian populations where data remain scarce. In addition, incorporating novel preventive strategies, such as letermovir prophylaxis, into future investigations will be crucial to assess its impact on CMV reactivation and transplant outcomes in this setting.

Importantly, while letermovir has emerged as a valuable prophylactic option in many high-income settings, it remains inaccessible in numerous transplant centers worldwide (39,40), including our own. In this context, our findings reinforce the effectiveness of vigilant viral load monitoring combined with timely preemptive therapy using ganciclovir or valganciclovir. These strategies remain critical tools for preventing CMV-related disease and mortality, especially in resource-limited environments where access to novel prophylactic agents is restricted.

Our findings highlight the persistence of CMV reactivation as a complication of allo-HSCT. The risk factors for CMV reactivation include underlying ALL, specific types of HLA matching (notably HLA-MMUD), and acute or chronic GVHD. Our analysis did not reveal a direct correlation between CMV reactivation and increased mortality, nor did it demonstrate a significant effect on patient survival following transplantation. These results underscore not only the complexity of CMV infection after allo-HSCT, but also the continued value of preemptive strategies in the absence of universal access to letermovir.

## Data Availability

Data will be made available on request.
